# The implication of non-AUG-initiated N-terminally extended proteoforms in cancer

**DOI:** 10.1080/15476286.2025.2498203

**Published:** 2025-04-25

**Authors:** Rita Pancsa, Dmitry E. Andreev, Kellie Dean

**Affiliations:** a Institute of Molecular Life Sciences, HUN-REN Research Centre for Natural Sciences, Budapest, Hungary; b Shemyakin-Ovchinnikov Institute of Bioorganic Chemistry, RAS, Moscow, Russia; c Belozersky Institute of Physico-Chemical Biology, Lomonosov Moscow State University, Moscow, Russia; d School of Biochemistry and Cell Biology, University College Cork, Cork, Ireland

**Keywords:** N-terminal extension, translation initiation, start codon, non-AUG initiation, alternative translation start site, proteoforms with altered N-termini, short linear motifs

## Abstract

Dysregulated translation is a hallmark of cancer, and recent genome-wide studies in tumour cells have uncovered widespread translation of non-canonical reading frames that often initiate at non-AUG codons. If an upstream non-canonical start site is located within a frame with an annotated coding sequence (CDS), such translation events can lead to the production of proteoforms with altered N-termini (PANTs). Certain examples of PANTs from oncogenes (e.g. c-MYC) and tumour suppressors (e.g. PTEN) have been previously linked to cancer. We have performed a systematic computational analysis on recently identified non-AUG initiation-derived N-terminal extensions of cancer-associated proteins, and we discuss how these extended proteoforms may acquire new oncogenic properties. We identified a loss of stability for the N-terminally extended proteoforms of oncogenes TCF-4 and SOX2. Furthermore, we discovered likely functional short linear motifs within the N-terminal extensions of oncogenes and tumour suppressors (SOX2, SUFU, SFPQ, TOP1 and SPEN/SHARP) that could provide an explanation for previously described functionalities or interactions of the proteins. In all, we identify novel cases where PANTs likely show different localization, functions, partner binding or turnover rates compared to the annotated proteoforms. Therefore, we propose that alterations in the stringency of translation initiation, often seen under conditions of cellular stress, may result in reprogramming of translation to generate novel PANTs that influence cancer progression.

## Introduction

A crucial step in protein synthesis is the selection of the translation initiation site on the mRNA. In eukaryotic cells, this process is largely accomplished through a scanning mechanism. The pre-initiation complex (PIC), which consists of the 40S ribosomal subunit, certain translation initiation factors, and a methionine-tRNA (met-tRNA), enters the 5’end of the mRNA and scans the mRNA sequence from 5’ to 3’. Recognition of a suitable start codon, typically AUG, leads to the dissociation of the initiation factors and the joining of the 60S subunit (reviewed in [1,2]). The ability of the PIC to reach and select a specific start codon on the mRNA depends on several factors, including the availability and activity of the translation initiation factors, the structure of the mRNA, and RNA-binding proteins. These factors can interfere with the scanning process and the selection of the start codon. Differences in mRNA sequences, which are inspected by scanning proteins, dictate translation factor dependency. While some mRNAs require a set of ‘canonical’ initiation factors for translation, others require additional non-canonical factors (reviewed in [3]). This allows for selective translation control, where alterations in signalling pathways differentially affect the translation of various mRNA species, resulting in different proteomic changes.

When the PIC (pre-initiation complex) scans the mRNA 5’ leader region, it encounters a potential start codon that can form a codon–anticodon interaction with the met-tRNA in the P site. The optimality of this interaction is probed by multiple components of the PIC, including rRNA bases, ribosomal proteins, and initiation factors. The efficiency of start codon recognition depends on several factors, including the identity of the codon (some non-AUG codons can also be recognized), its nucleotide context, and the presence of certain initiation factors, such as eIF1, eIF1A, and eIF5. Due to these factors, the scanning ribosome can continue scanning if the start codon is not optimal, a phenomenon known as leaky scanning [4]. Leaky scanning means that translation initiation can occur at more than one start codon on certain mRNAs.

If these start codons are located within the same open reading frame and upstream of each other, translation can produce polypeptides with extended N-termini [5,6] which may yield alternative proteoforms. The term ‘proteoform’ describes all molecular variants in which a protein can exist and is typically used to refer to differences at the protein level (i.e. translational products). While isoforms are typically produced by alternative splicing of the mRNAs, proteoforms can be produced by translational and/or post-translational mechanisms, such as alternative translation initiation, stop codon readthrough, posttranslational modifications or proteolytic processing. Although we cannot precisely predict all the posttranslational mechanisms, and thus the actual proteoforms that will exist in cells, we refer to the N-terminally extended polypeptides as proteoforms because they differ from the canonical forms on the level of translation.

Deregulated translation is a hallmark of cancer cells [7–14]. Regarding non-AUG initiation events, there are numerous examples of N-terminally extended proteoforms for genes involved in cancer progression, one of the earliest such discoveries was made for the *c-MYC* oncogene in 1988 [15]. By analysing the *in vitro* protein-coding capacity of *c-MYC* cDNAs, Hann et al. found that c-MYC1 (p67) proteoform was initiated by an upstream CUG codon, adding a 14-amino acid extension; whereas the c-MYC2 (p64) proteoform was initiated by the canonical AUG start [15]. While there was lower translation efficiency of *c-MYC1*, with total synthesis about 10–15% of the level of the canonical protein, this changed when cells were grown to high densities, and this effect seemed to be due to amino acid restriction [16,17]. Overall, early studies on *c-MYC* translation pointed to modulation of the scanning ribosomal pre-initiation complex that impacted start codon selection, resulting in the production of different MYC proteoforms. More recently, work by Sato *et al*. (2019), showed that the balance of c-MYC proteoforms could be tipped in favour of the AUG-initiated isoform by the oncogenic protein, 5MP1 (eukaryotic initiation factor 5 (eIF5)-mimic protein) that competes with eIF5 [18] and promotes malignancy in colorectal cancer by translational reprogramming [19].

In a cancer context, tumour suppressor proteins act as negative regulators in biochemical and cellular processes, essentially serving as brakes within a system. Indeed, loss or disabling mutations of tumour suppressor genes, like *RB1*, *TP53* and *PTEN*, are found in numerous cancers [20]. The phosphatase and tensin homolog on chromosome ten, *PTEN*, encodes a protein that blocks the activation of the phosphatidylinositol 3-kinase (PI3K)/AKT pathway by dephosphorylating lipid substrates [21,22], thereby influencing cell proliferation, survival, growth and metabolism.

The first description of N-terminal extension of PTEN came from a systematic analysis of the extent of non-AUG initiation in human [23]. Ivanov et al. (2011) proposed that PTEN had a 173-aa extension due to translation initiation from a CUG codon located 519 nucleotides upstream of the canonical AUG start [23]. The extended form was supported experimentally and termed PTEN-L (PTEN-Long or PTEN-a) [24–26]. The PTEN family was further expanded by another N-terminal extended proteoform that is initiated by an in-frame AUU codon (PTEN-M or PTEN-b), adding 146 aa [26,27]. Taken together, N-terminally extended proteoforms of PTEN have been shown to have altered localization, substrates and interaction partners [25,27,28].

More recent work suggests that PTEN translational variants can modify gene expression by promoting histone methylation, opposing PTEN’s traditional role as a tumour suppressor [29]. Through a direct interaction with WD40 repeat-containing protein 5, WDR5, the extended PTEN proteoforms can modulate the efficiency of histone H3K4 methyltransferases, which in turn, upregulates expression of target genes such as *Notch3* [29]. Binding studies and a crystal structure (PDB:8X3S) showed that the N-terminal extension of PTEN-a contains a WDR5-interacting motif (WIN; aa116–148) [30], wherein mutagenesis of the key residues effectively reduced the protein’s pro-proliferative effect in different tumour models [30]. Besides conferring new functionality to PTEN, the N-terminal extension of PTEN is also responsible for altered protein stability due to interactions with a substrate recognition component of the SCF (SKP1-CUL1-F-box protein) E3 ubiquitin-protein ligase complex, mediating ubiquitination [29].

Cancer-associated genes can have conflicting roles as oncogenes and tumour suppressors, depending on the specific context and cancer type. An example is Wilms’ tumour, WT1, a transcription factor that has an important role in developmental processes and cell survival [31]. *WT1* was first identified as a tumour suppressor gene in Wilms’ tumours [32] but subsequent work showed that overexpression or mutation of WT1 contributes to tumorigenesis of some leukaemias and solid tumours [33,34]. For many years, how WT1 both promotes and suppresses cancers remained elusive [35]. However, recent work indicates that WT1 with a CUG-initiated, 68-aa N-terminal extension is the oncogenic form of the protein, cugWT1 [36,37]. Lee et al. found that cugWT1 extension was phosphorylated at S62 by AKT, leading to increased stability of WT1 and increased expression of its cancer-promoting target genes [36]. Subsequent work supported an oncogenic role of cugWT1 in mouse models of colorectal and lung cancers [37].

All cells rely on growth factors to mediate cell proliferation, differentiation, survival and migration, and dysregulation of growth factor signalling often contributes to cancer [38]. N-terminally extended proteoforms also impact growth factor signalling as illustrated by fibroblast growth factor 2 (FGF2, also known as basic FGF or bFGF) and vascular endothelial growth factor (VEGF). For FGF2, four non-AUG initiated forms of the protein were discovered, resulting in proteins of 22, 22.5, 24 and 34kDa [39–41]. Within the N-terminally extended versions, a conserved glycine-arginine repeat motif with several methylated arginine residues has been found to promote nuclear transport and/or retention [42–44].

Similar to FGF2, VEGF also has several extended proteoforms that can be generated from initiation at upstream, in-frame CUG codons [45,46], with the longest proteoform having a 180-aa N-terminal extension (VEGF-L). VEGF-L is subject to proteolytic processing [47], and in recent work, Katsman *et al*. (2022) provide evidence that the resulting proteolytic fragment, N-VEGF, translocates to the nucleus to participate in transcriptional regulation of angiogenic genes, including *VEGF*, and key genes associated with cell survival under hypoxic conditions [48].

The development of ribosome profiling techniques has allowed researchers to discover novel translated regions in the human genome [49,50]. Recently, Fedorova and colleagues performed global analyses of Ribo-seq data and phylogenetic conservation, uncovering thousands of novel N-terminal protein extensions encoded by human mRNAs [51]. We reasoned that some of these novel N-terminal extensions may be involved in cancer. Our study explores N-terminal extensions of cancer-related proteins and proposes different ways in which they may provide differential intracellular localization, stability, and interactions.

## Results

**Figure 1.**
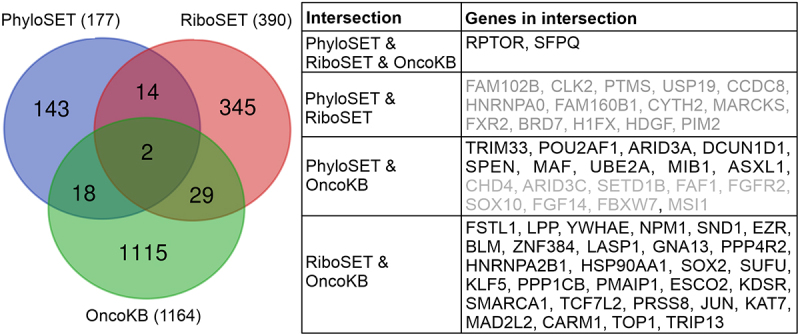
The Venn diagram shows the overlaps between the two datasets of genes encoding N-terminally extended proteins obtained from Fedorova et al., PhyloSET (177 genes) and RiboSET (390 genes), and the list of cancer-associated genes from OncoKB (1164 genes). On the right, genes belonging to the different intersections of the Venn diagram are listed. Cancer-associated genes with experimentally supported N-terminal protein extensions that are in the focus of this study are listed in black, while genes that were excluded due to not being annotated in OncoKB (intersection of PhyloSET & RiboSET) or showing no sign of translation upstream of the annotated start codon by RiboCrypt (intersection of PhyloSET & OncoKB, second half of gene list) are listed in grey.

Fedorova et al. [51] performed two types of analysis. First, they generated a set of genes with translated N-terminal extensions called RiboSET using aggregated Riboseq data. Second, they carried out an analysis of the evolutionary conservation of N-terminal extensions to show evidence of protein coding evolution. Genes with these evolutionary conserved N-terminal extensions were included in PhyloSET. We decided to find which genes from RiboSET and PhyloSET were known in relation to cancer. Analysis of OncoKB [52,53] yielded 49 cancer-related genes with potential N-terminal extensions (Figure 1). Manual annotation of the translated N-terminal extensions of 18 PhyloSET-derived genes using RiboCrypt allowed us to predict translated N-terminal extensions in 9 cases (see Methods). Therefore, our computational analysis was performed on 40 cancer-associated genes with experimentally supported N-terminal extensions (Figure 1; Supplementary Table S1).

We have considered several different scenarios regarding how N-terminal extension can influence protein availability, activity, or novel function. First, N-terminal extensions can affect proteoform stability. To investigate this possibility, we have compared the predicted stabilities of proteoform pairs using the Degronopedia resource [54]. Second, the N-terminal extension can either add a signal sequence or interfere with an existing signal sequence to alter protein targeting. This was explored using SignalP 6.0 [55]. Third, the N-terminal extension could potentially alter the folding of a proteoform, which can influence or even completely change its activity. For selected cases, we used AF3 [56] to predict the structure of N-terminally extended proteoforms. Finally, the N-terminal extension can obtain short linear motifs (SLiMs/ELMs) that can add novel binding partners, sites for posttranslational modifications, and localization signals. To do this, we explored N-terminally extended proteoforms using the ELM database, which is a resource containing a collection of annotated eukaryotic linear motifs [57].

When investigating the altered stabilities of proteoforms, we consider two possible scenarios. If an N-terminally extended proteoform is more stable than an AUG-initiated proteoform, then increasing initiation at a non-AUG codon could significantly increase the level of the protein. Conversely, if an N-terminal extension makes a proteoform less stable, then increasing non-AUG initiation would rapidly deplete protein levels. Our analysis using Degronopedia shows that for 6 proteins (UBE2A, LPP, SND1, CARM1, TCF7L2 and SOX2) the extended form is considerably less stable (at least 1 unit on the predicted protein stability index (PSI) scale) than the annotated form. Among these, the largest reduction in stability is observed for the TCF7L2/TCF-4 tumour suppressor (more than 2 PSI units) that regulates the WNT signalling pathway and transactivates downstream target genes involved in the progression of colorectal cancer. It is directly involved in regulating the expression of the oncogene MYC and inducing epithelial-mesenchymal transition (EMT) [58–61]. At the same time, there are two proteins, FSTL1 and PRSS8, for which the extended form is considerably more stable (at least 1 unit on the PSI scale) than the annotated form (Table S1). In the case of 12/40 cancer-associated proteins the N-terminally extended form is also predicted to alter the cleavage status of the initiator methionine. For 8 proteins the Met is predicted to be cleaved in the normal form, while not cleaved in the extended form, while for 4 proteins it is the other way around (Table S1).

Next, we investigated whether N-terminal extensions could contribute to signal sequence recognition. Although none of the extensions added novel signal sequences, in two genes, N-terminal extensions may interfere with protein secretion and translocation. For FSTL1, the N-terminal extension significantly decreased the probability of protein targeting (0.5716 vs 0.9998). In contrast, for PRSS8, the N terminus was predicted to preclude targeting. PRSS8 has been described as a potent tumour suppressor in colorectal carcinogenesis and metastasis [62] and is normally secreted to the extracellular space to be part of the seminal fluid. Increased expression of the N-terminally extended, likely mis-targeted proteoform, at the expense of the canonical one, could reduce the availability of PRSS8 in the seminal fluid and thereby contribute to cancer progression.

As a next step, we selected cases for structural modelling by AF3 [56] to identify structural changes introduced by the extensions. We were primarily interested in the cases where the extension could interfere with homo-oligomerization or heterodimerization, so we selected a subset of the 40 proteins with such information in UniProt [63](see Table S1 column U). While there are likely structural changes introduced by the N-terminal extensions, in our tested cases, the AF3 structural predictions did not show consistent changes between the five models produced for WT and five models representing extended forms. Often the five models obtained for the WT form already showed large structural variability (based on visual inspection in ChimeraX), which precluded the detection of structural changes caused by the extensions. Despite considerable effort put into this part of the analysis, we found the data to be too inconclusive to report any results.

Finally, we explored how SLiMs in N-terminal extensions could affect the function of proteins. The extended proteoforms were searched for linear motif candidates using the ELM database as a web server. The resulting hits were filtered for motifs overlapping with the extensions (cleavage motifs and overly redundant motifs with a probability score > 0.015 were removed; see candidate extension motifs per protein in Table S1). Although this search resulted in a plethora of candidate motifs, most of which are likely not true, functional linear motifs, we believe that careful analysis of such information on a case-by-case basis including functional profiling, literature mining and consideration of the known interaction partners of the investigated proteins could result in the discovery of new protein functional modules. Therefore, we decided to conduct targeted analyses of selected candidates and present our results in the next section of the manuscript.

## SFPQ

Splicing factor, proline- and glutamine-rich, SFPQ (Uniprot P23246, also known as PSF; 707 aa) is a predominantly nuclear matrix-associated, nucleic acid-binding protein that is one member of the *Drosophila behavior/human splicing* (DBHS) family of proteins [64]. Although the protein was first identified through its participation in messenger RNA (mRNA) splicing as a component of the spliceosome and U4/U6.U5 small nuclear ribonucleoprotein (tri-snRNP) complexes [65–67], it is clear that SFPQ mediates many nuclear events in addition to splicing [64].

SFPQ has an N-terminal disordered region that is enriched with proline and glutamine residues, a DNA-binding domain (DBD), two RNA-recognition motifs (RRM1 and RRM2), and a protein interaction domain (NonA/paraspeckle (NOPS)) that includes an extended coiled-coil subdomain that is important for dimerization, as shown in the resolved crystal structure (PDB: 4WII [68]). SFPQ has a nuclear localization sequence (NLS) at its C-terminus (aa701–707); however, there are reports that the protein has non-nuclear roles in the cytoplasm [69,70].

One of SFPQ’s main protein-binding partners is non-POU domain-containing octamer-binding protein, NONO, a multifunctional, RNA-binding protein that is also a DBHS family member [71,72]. SFPQ and NONO can be arranged as a heterodimer and a crystal structure has been resolved (PDB: 7PU5 [73]). SFPQ and NONO can also form a ternary complex with topoisomerase I, TOP1 (see below) that stimulates the enzyme’s activity [74,75].

Given a wide range of cellular activities, it is perhaps unsurprising that SFPQ has been implicated in several human diseases, including neurological disorders and cancer [76]. Knockdown of SFPQ in colorectal cancer cells was shown to enhance apoptosis [77], and multiple reports link SFPQ to altered transcriptional, transport and splicing profiles of mRNAs involved in cancer progression and drug resistance [78–80]. Still the most direct involvement in cancer comes from Xp11.2 translocation renal cell carcinoma (XP11.2 tRCC) in which *SFPQ* is the fusion partner of MiT (microphthalmia transcription factor) family member gene, transcription factor binding to IGHM enhancer 3 (*TFE3*) [81]. The *SFPQ/TFE3* gene fusion is also present in perivascular epithelioid cell tumours (PEComas) and melanotic Xp11 translocation renal cancers [82].

**Figure 2.**
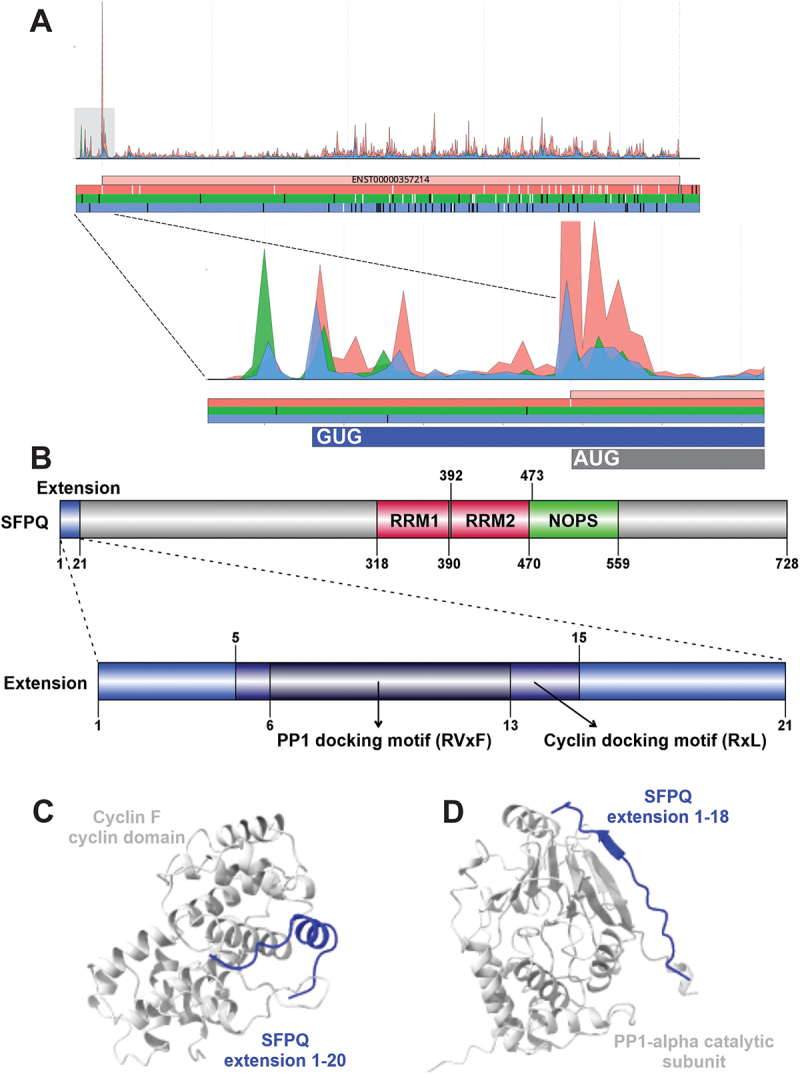
(A) Aggregated Riboseq data for *SFPQ* (ENST00000357214) from the Ribocrypt transcriptome browser (ribocrypt.Org, in preparation). Ribosome footprints are colour-coded according to the translated reading frames shown below the diagram. (B) Domain map of the N-terminally extended SFPQ protein is depicted. The 21 aa extension (blue) and the 707 aa canonical protein are shown, with known domains of the protein depicted (residue boundaries provided with respect to the extended proteoform). Below, the extension is depicted separately with the two identified, likely functional short linear motifs marked by dark blue and black, respectively. (C) AlphaFold 3 (AF3) models of the proposed domain-motif complexes are provided below the domain maps in cartoon representation. Structural model of human cyclin F cyclin domain (P41002, residues 290–531) modelled with SFPQ extension peptide residues 1–20, containing the predicted cyclin-binding RxL motif. (D) Structural model of human Serine/threonine-protein phosphatase PP1-alpha catalytic subunit (P62136, residues 1–305) modelled with SFPQ extension peptide residues 1–18, containing the predicted PP1 docking RVxF motif.

The N-terminal extension of SFPQ is supported by strong ribosome footprint densities within the region upstream of the annotated start codon (Figure 2(A)). To search for proteomic evidence of the N-terminal extension of SFPQ, we implemented targeted peptide search engine PepQuery2 [83]. The search in 48 MS/MS datasets available in the web version (https://pepquery2.pepquery.org/) yielded 71 confident peptide spectrum matchings (PSMs) corresponding to 3 peptides: FCLDRPLTTDMSR (64×), MASTFPER (3×), MASTFPERLLR (4×) (Supplementary Table S2). In the 21-residue N-terminal extension of SFPQ we could identify two unique motifs that likely confer new protein–protein interactions (Figure 2(B)). The first is DOC_CYCLIN_RxL_1 (aa5–15, FPERLLRFCLD), a cyclin N-terminal domain docking motif. Work by Rayner et al. (2021), using a proximity ligation method (*Bio*tin *Id*entification (BioID)), immunoprecipitations and mass spectrometry, identified novel interaction partners of cyclin F, including SFPQ [84]. This interaction was further supported by high-throughput data [85].

Although not examined in a cancer context, an interaction between cyclin F and SFPQ is of clinical relevance to familial and sporadic amyotrophic lateral sclerosis (ALS) and frontotemporal dementia (FTD). In 2016, mutations of the cyclin F gene, *CCNF*, were discovered in ALS/FTD, and *CCNF* variant (cyclin F: S621G) resulted in an accumulation of ubiquitinated proteins, likely due to abnormal ubiquitination or transport to the proteasome [86]. SFPQ was already linked to pathological features of neurological disorders, including ALS [87]. Following on from this, it was shown that overexpression of the cyclin F mutant S621G led to an increase in the insoluble fraction of SFPQ and disrupted its subcellular distribution [84]; thus, cyclin F mutations could lead to dysregulation of SFPQ’s influence on RNA metabolism, which contributes to the pathomechanisms of ALS/FTD.

It is possible that the RxL motif in the N-terminal extension might mediate this interaction, as there is no other copy of this motif within SFPQ. Although cyclin F is more of an E3 ligase adaptor than an ordinary cyclin [88], it has cell cycle regulatory activity under certain circumstances and a cyclin domain that was shown to be functional and binding to RxL motifs (also called Cy motif) in several proteins [89–91]. Modeling of the interaction by AF3 led to a complex wherein the RxL motif of the SFPQ extension binds to the known RxL-binding groove of the Cyclin F cyclin domain in helical conformation, similarly to RxL motif complexes with known structure (e.g. PDB: 1H24, 1H25, 1H26, 1H27, 1H28 [92]) (Figure 2(C)).

The second motif identified in the N-terminal extension is DOC_PP1_RVXF_1 (aa6–13, PERLLRFC), which overlaps with the RxL motif. The RVxF motif mediates the interaction with serine/threonine-protein phosphatase 1, PP1 [93]. Previous data provide evidence for an interaction between SFPQ and PP1. SFPQ was found to interact with PP1CA (the catalytic subunit of the PP1 phosphatase) in a low- and high-throughput study [94]. While for the close-relative NONO, the RVxF motif could be identified and was determined to mediate an interaction with PP1, it could not be found in SFPQ [94].

We suggest that the RVxF motif within the N-terminal extension of SFPQ could be functional and mediating the interaction with PP1, which could be especially important when SFPQ forms homodimers and does not form a complex with NONO. In the AF3-predicted complex the RVxF motif of the SFPQ extension binds to PP1-alpha catalytic subunit in beta-augmentation, similarly to other bound RVxF motifs with known structure (e.g. PDB: 3N5U [95]) (Figure 2(D)). This interaction could lead to dephosphorylation of SFPQ and loss of its transcriptional co-repressor activity [94].

## SPEN

The SHARP protein encoded by gene SPEN is a huge transcriptional repressor of 3664 residues that acts as a scaffold for different proteins and complexes important for expression regulation. It was identified as a component of transcriptional repression complexes in both nuclear receptor and Notch/RBP-Jkappa signalling pathways. The N-terminal ~ 600 residues contain 4 RRM domains that were reported to mediate DNA as well as RNA binding, and the protein has extended disordered regions that exceed thousand residues in length. A central RID domain was reported to bind nuclear receptors, while the C-terminal SPOC domain is known to recruit transcriptional corepressors SMRT/NCoR and histone deacetylases HDAC1/2 through binding their LSD motifs [96–98], as well as the C-terminal disordered domain of RNA polymerase II (Pol II [99]). The RBP-Jkappa/SHARP complex was shown to recruit the CtIP and CtBP corepressors to silence Notch target genes in a manner that CtIP/CtBP functionally complement SHARP in repression [100]. While CtIP binding could be mapped to the repressor domain of SHARP, CtBP binding could not be precisely mapped.

**Figure 3.**
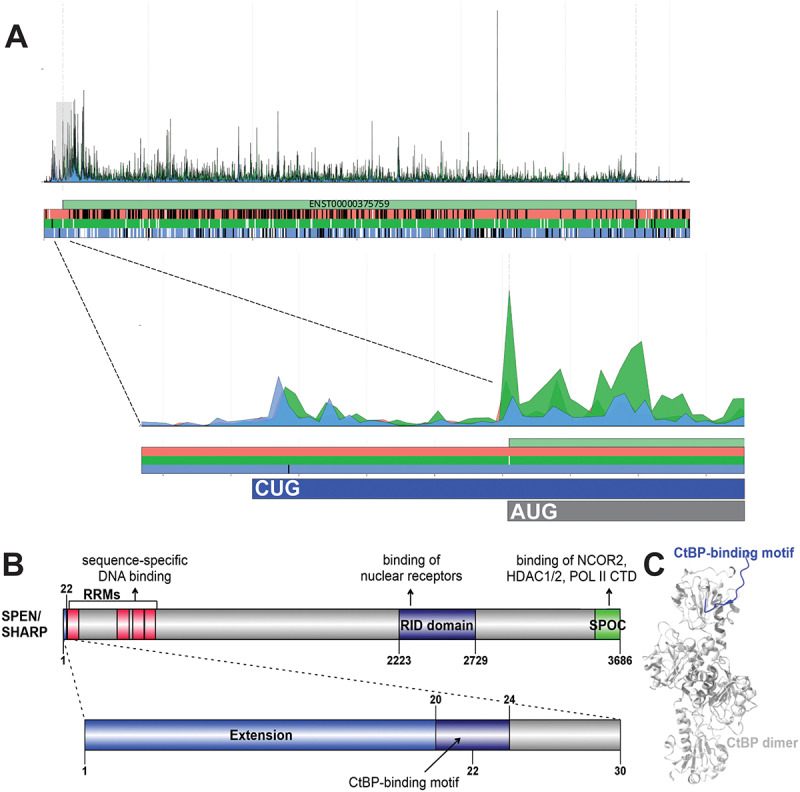
(A) Aggregated Riboseq data for *SPEN* (ENST00000375759) from the Ribocrypt transcriptome browser (ribocrypt.Org, in preparation). Ribosome footprints are colour-coded according to the translated reading frames shown below the diagram. (B) Domain map of the N-terminally extended SPEN/SHARP protein is depicted. The 22 aa extension (blue) and the 3664 aa canonical protein are shown, with known domains of the protein depicted (residue boundaries provided with respect to the extended proteoform). Below, the extension is depicted separately with the identified, likely functional CtBP-binding motif marked by dark blue. (C) the AF3 model of the proposed domain-motif complex between human CtBP1 dimer (2× P41002, residues 28–370) and the SHARP extension peptide residues 15–30 (containing the predicted CtBP-binding motif) is provided on the right of the domain maps in cartoon representation.

However, translation from an in-frame CUG codon in 5’leader leads to a 22 aa-long N-terminal extension (Figure 3(A)), which is supported by PepQuery2 proteomics analysis uncovering 6 confident peptide spectrum matchings (Table S2). Interestingly, this short extension of SHARP contains a CtBP-binding motif, while the annotated fraction of the giant protein does not, which makes it highly likely that the extended form plays a key role at least in the silencing of Notch genes, where CtBP binding is required (Figure 3(A,B)). Modeling of the interaction by AF3 resulted in a complex structure wherein the SHARP CtBP-binding motif binds to the same groove of the CtBP dimer with beta-augmentation as seen for known CtBP-motif complexes (e.g. PDB:1HL3 [101]) (Figure 3(C)). Thus, the extension might contribute to CtBP binding simultaneously to CtIP binding by the annotated part of the protein, and/or could enable direct binding of SHARP to CtBP without the contribution of CtIP in certain processes.

## SUFU

Suppressor of fused homolog, SUFU (Uniprot Q9UMX1; 484 aa), is a negative regulator within the evolutionarily conserved Hedgehog (HH) signalling pathway that is fundamental in embryogenesis and adult tissue homoeostasis [102]. SUFU interacts with zinc finger GLI transcription factors (GLI1, GLI2 and GLI3) in the cytoplasm [103,104], preventing their translocation to the nucleus to activate transcriptional programs. However, it was also shown that SUFU can interact with GLI1 while bound to DNA [104]. SUFU N-terminal domain (aa 64–240) binds to the C-terminal region of GLI; while SUFU_C (aa 253–473) binds to the N terminal of GLI, and interactions between SUFU and GLI1 at both sites are required for cytoplasmic tethering and repression of GLI1 [105]. The repressive effect of SUFU on GLI transcription factors can be overcome by serine-threonine kinase, Fused (serine-threonine kinase protein-36, STK36 in humans) [106].

Disruption of the HH pathway is linked to cancer development and components of the pathway are targets for anticancer therapeutics [107]. In humans, germline and somatic mutations in *SUFU*, accompanied by loss of heterozygosity (LOH), results in a predisposition to medulloblastoma [108], a type of brain cancer most often affecting children. Subsequent work in mouse models showed that *Sufu* ^±^ mice crossed within a *p53* null background led to a stark increase in medulloblastoma in the animals [109]. SUFU has also been implicated in other cancers, including basal cell carcinoma [110]. In somatic cells, missense mutations across the protein are most common, with 27.94% of 1124 unique samples containing *SUFU* missense mutations recorded in COSMIC (cancer.sanger.ac.uk) [111].

SUFU is normally targeted for degradation by the Skp1-Cul1-F-box protein complex (SCF) through its polyubiquitination by E3 ubiquitin ligase, FBXL17 (F-box and leucine-rich repeat protein 17), while in complex with GLI1 [112]. Although FBXL17 directs downregulation of SUFU in the nucleus [112], it does not seem to do this in a cell cycle-dependent fashion, due to the lack of cyclin-dependent kinase (CDK) phosphorylation sites in SUFU. More recent work has shown that SUFU can operate outside of its role in HH signalling by negatively regulating initiation of centrosome duplication and DNA replication at the G1-S transition of the cell cycle [113].

**Figure 4.**
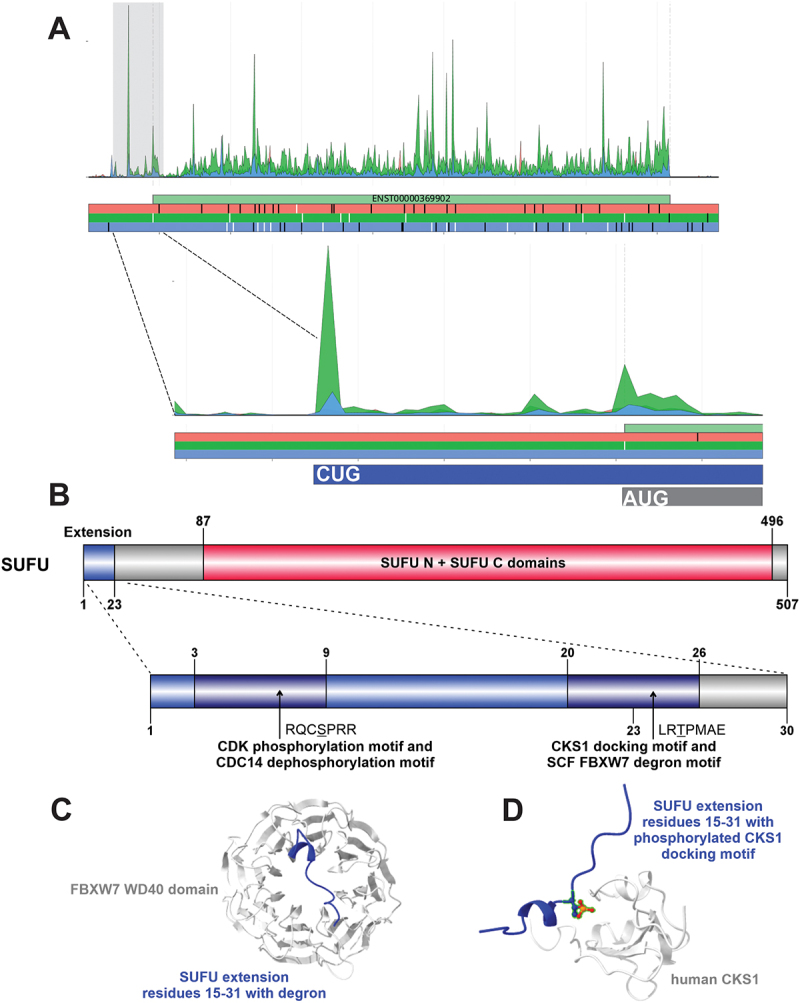
(A) Aggregated Riboseq data for *SUFU* (ENST00000369902) from the Ribocrypt transcriptome browser (ribocrypt.Org, in preparation). Ribosome footprints are colour-coded according to the translated reading frames shown below the diagram. (B) Domain map of the N-terminally extended SUFU protein is depicted. The 23 aa extension (blue) and the 484 aa canonical protein are shown, with known domains of the protein depicted (residue boundaries provided with respect to the extended proteoform). Below, the extension is depicted separately with the four identified, likely functional short linear motifs mapped onto two regions (in dark blue), respectively. (C) AF3 models of two of the proposed domain-motif complexes are provided below the domain maps in cartoon representation. Structural model of human FBXW7 WD40 domain (Q969H0, residues 370–707) bound to SUFU extension peptide residues 15–31, containing the predicted degron in a phosphorylated form (p22T). (D) Structural model of human CKS1 (P61024, residues 1–79) modelled with SUFU extension peptide residues 15–31, containing the predicted CKS1 docking motif in a phosphorylated form (p22T). The phosphorylation is highlighted in stick representation. For the identified modification-type motifs, complexes could not be modelled due to the transient nature of the interactions and lack of available experimentally determined complex structures.

Analysis of Riboseq datasets allowed the detection of translation of CUG-initiated N-terminal extension (Figure 4(A)), which is also supported by PeptideQuery2-based identification of 5 confident PSMs (Table S2). Based on our subsequent motif analysis, the N-terminal extended form of SUFU could be under the control of the cell cycle (Figure 4(B)), as suggested by the joint presence of several motifs implicated in granting cell cycle phase-dependent availability. Efficient phosphorylation by CDKs at motifs (MOD_CDK_SPK_2, MOD_CDK_SPxK_1) could be largely fostered by the observed cyclin-dependent kinases regulatory subunit 1 (CKS1) docking motif (DOC_CKS1_1). The sequence pattern of the CDK phosphorylation site also matches the target sequence pattern required for dephosphorylation by the CDK-antagonistic phosphatase, CDC14 (MOD_CDC14_SPxK_1). Furthermore, on the border of the extension and the canonical protein sequence, a DEG_SCF_FBW7_2 motif is also formed. The SCF ubiquitin ligase substrate recognition subunit F-box/WD repeat-containing protein 7, FBXW7, is a well-known tumour suppressor, initiating the ubiquitination and subsequent degradation of many cell cycle-regulated proteins, including oncogenes such as *CCNE1*(cyclin E), *MYC* and *RICTOR* [114–116]. In the AF3-predicted model of the SUFU-FBXW7 interaction, the SUFU motif is placed onto the top of the doughnut-shaped WD40 domain of FBXW7 in a similar conformation as seen for other FBXW7 substrates (e.g. PDB:2OVQ [117]) (Figure 4(C)). Modeling of the potential SUFU-CKS1 interaction resulted in a complex, where the clam-shaped CKS1 binds the SUFU extension peptide (phosphorylated at T22) in a similar conformation as seen for other CKS1-phosphopeptide complexes (e.g. PDB:2AST [118]) ((Figure 4(D)). Presence of all these motifs conferring cell-cycle dependent control within the relatively short, 23-residue disordered extension of SUFU can be considered as a solid support for the existence of a cell cycle-regulated SUFU proteoform.

## SOX2

One example of N-terminal extension is found in *SOX2*. The high-mobility group (HMG) box transcription factor, SOX2, is a master regulator of stemness and pluripotency. By promoting oncogenic signalling and maintaining cancer stem cells, SOX2 is implicated in the development of several different cancer types, including breast [119], prostate [120,121], pancreatic [122], gastric [123], lung [124] and cervical cancers [123]. SOX2 was proposed to promote metastasis [125–128], and its amplification was observed in several cancer types [125,129–135].

**Figure 5.**
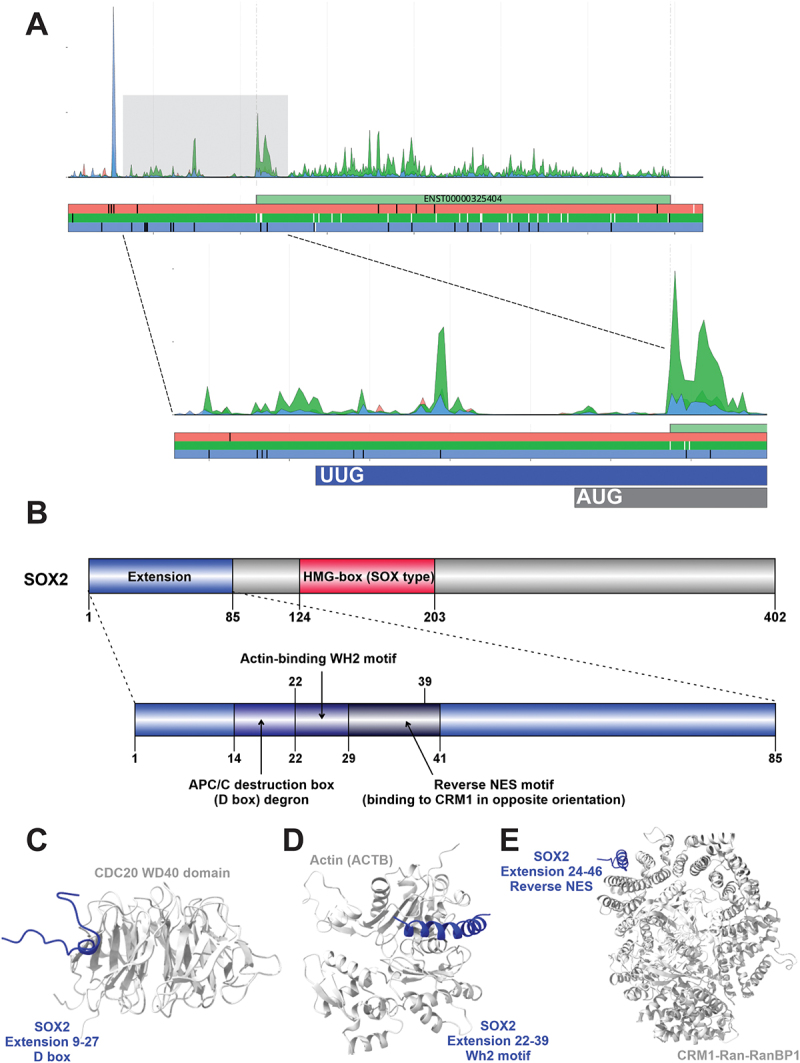
(A) Aggregated Riboseq data for *SOX2* (ENST00000325404) from the Ribocrypt transcriptome browser (ribocrypt.Org, in preparation). Ribosome footprints are colour-coded according to the translated reading frames shown below the diagram. (B) Domain map of the N-terminally extended SOX2 protein is depicted. The 85 aa extension (blue) and the 317 aa canonical protein are shown, with known domains of the protein depicted (residue boundaries provided with respect to the extended proteoform). Below, the extension is depicted separately with the three identified, likely functional short linear motifs marked by dark blue and black, respectively. (C) AlphaFold 3 (AF3) models of the proposed domain-motif complexes are provided below the domain maps in cartoon representation. Structural model of human CDC20 WD40 domain (Q12834, residues 160–477) modelled with SOX2 extension peptide residues 9–27, containing the predicted APC/C D box motif. (D) Structural model of human cytoplasmic actin (P60709, residues 1–375) modelled with SOX2 extension peptide residues 17–45, containing the predicted actin-binding WH2 motif. (E) structural model of a CRM1-ran-RanBP complex (chains A, B and C of the PDB:5DIF structure were used) modelled with SOX2 extension peptide residues 24–46, containing the predicted reverse NES motif.

Aggregated Riboseq data for *SOX2* is consistent with translation of an N-terminal extension from the UUG codon located 288nts upstream of the annotated AUG start codon (Figure 5(A)). PepQuery2 analysis [83] allowed identification of as many as 90 peptide spectrum matches, corresponding to several unique peptides including AGPAHSAR (23X), MITIIGGGR (18X), MITIIGGGRIGQR (22X) and LPSSSPPAR (11X) (Table S2). Identification of semi-tryptic peptides started with methionine (MITI …) supports the assumption that the UUG codon of SOX2 N-terminal extension is decoded with initiator met-tRNAi.

The sequence of the predicted N-terminal extension was analysed with the eukaryotic linear motif (ELM) resource [57] and yielded 36 motifs that met our criteria (see Methods and Table S1). Interestingly, one of such motifs is a putative reverse nuclear export signal (NES; ELM class: TRG_NESrev_CRM1_2) located in the middle of the N-terminal extension (Figure 5(B)). While as a transcription factor, SOX2 is expected to operate only in nuclei, a cytosolic pool of SOX2 also exists [136,137]. Cytosolic SOX2 was reported to interact with ribosomes and to regulate the translation of mRNAs that code for proteins implicated in sugar metabolism, matrix contacts, and development [137]. While the annotated sequence of SOX2 has a known NES that needs to get acetylated (at K75, probably by CBP/p300) for mediating the nuclear export of SOX2 [138], the extended isoform could potentially ensure a cytoplasmic pool of SOX2 independent of acetylation. Thus, decreased stringency of start codon selection has the potential to change the ratio of cytoplasmic and nuclear SOX2 to reprogram gene expression.

Additionally, the 85 residues long extension of SOX2 also contains a predicted Anaphase promoting complex (APC/C) D box degron motif (Figure 5(B)). This is likely functional, as one of the substrate recognition/activator subunits of the APC/C that can recognize this motif, CDH1/FZR1, is a SOX2 binding partner [139], and because for CDC20 (the other D box-binding activator subunit), a CDC20-APC/SOX2 signalling axis has been proposed to control some key biological properties of glioblastoma stem cells [140]. Since other binding modules enabling binding to the APC/C do not seem to be present in the annotated SOX2 sequence, it is likely that the D box motif of the extension mediates these interactions.

Furthermore, the extension contains a predicted actin-binding WH2 motif (Figure 5(B)), which could also be functional. We have analysed previously performed pull-down assays of SOX2 from the cell lysates of three different cell types [137,140] and found that SOX2 bound cytoplasmic actin (ACTB) in all three cell types along with some proteins involved in the regulation of actin filament assembly, such as cofilin and actin capping protein. The presence of this rather complex, low-probability actin-binding motif and consistently detected SOX2–actin interactions suggest a hitherto undiscovered function of cytoplasmic SOX2 in binding to or regulating the actin cytoskeleton.

We have performed AF3 predictions of the three identified SOX2 extension motifs with the respective motif-binding domains (CDC20 WD40 domain for the D box, Actin for the WH2 motif and the CRM1-Ran-RanBP1 complex for the reverse NES), which resulted in complex structures (Figure 5(C,D,E)) where the SOX2 motifs were bound to the right surface patch/binding groove of the interacting domains with respect to the experimentally determined structures of the same motif-domain interactions (e.g. PDB:4UI9,8A2T [141,142] and PDB:4BH6 [143]; PDB:5YPU [144] and PDB:5DIF [145] for the three motifs, respectively).

## TOP1

**Figure 6.**
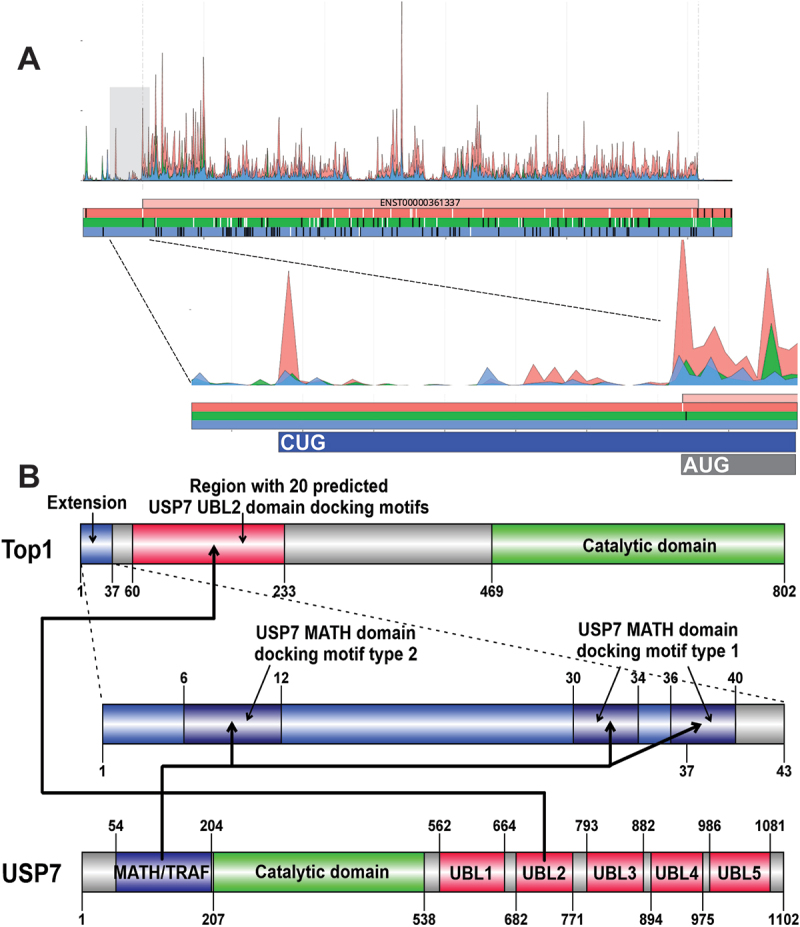
A) Aggregated Riboseq data for *TOP1* (ENST00000361337) from the Ribocrypt transcriptome browser (ribocrypt.Org, in preparation). Ribosome footprints are colour-coded according to the translated reading frames shown below the diagram. B) at the top, the domain map of the N-terminally extended TOP1 protein is depicted. The 37 aa extension (blue) and the 765 aa canonical protein are shown, with known domains of the protein depicted (residue boundaries provided with respect to the extended proteoform). Below, the extension is depicted separately with the three identified, likely functional USP7 MATH domain-binding short linear motifs marked by dark blue. At the bottom, the domain map of USP7 is shown, with the domain boundaries provided based on Figure 1 of Valles et al. [146]. Thick black arrows connect the domains of USP7 with the corresponding binding motifs in extended TOP1 that likely interact. The interactions could not be modelled by AF3, because it could not correctly predict the beta-rich domain structure of the USP7 MATH domain.

Analysis of Topoisomerase I, TOP1 allowed identification of CUG-initiated N-terminal extension (Figure 6(A)) which is supported by 30 PSMs identified with PepQuery2 (Table S2). TOP1 is an enzyme that can relax positive and negative supercoiling as well as torsional tension of the DNA induced by DNA replication and transcription. While performing this task, TOP1 creates single strand breaks (SSBs) that allow relaxation of the torsional tension of DNA and forms covalent DNA-protein crosslinks (DPCs), specifically TOP1 cleavage complexes (TOP1ccs) [147]. DPCs represent a double-edged sword as they are necessary for genome maintenance, but at the same time, when they accumulate due to deregulated removal, they can be detrimental for the cell due to blocking replication and transcription, as well as other processes involving DNA. TOP1ccs are among the most frequently occurring DPCs and therefore even anticancer drugs were developed that aim to kill cancer cells by trapping TOP1ccs [147].

The repair of TOP1ccs is regulated on one hand by the PARylation-dependent TDP1 pathway, wherein TDP1 is capable to cleave the covalent bond between TOP1 and DNA [148], and the ubiquitylation-dependent proteasome pathway that mediated TOP1 degradation [149]. PARylation of the TOP1ccs was demonstrated to enhance USP7-mediated deubiquitination [150], indicating that USP7 can probably reverse RNF4-induced ubiquitylation of TOP1-DPCs [150,151].

The annotated, N-terminal disordered region of TOP1 contains 20 predicted USP7 ubiquitin-like 2 (UBL2) domain docking sites, wherein many lysins involved in the predicted docking motifs (described as KxxxK) are known sumoylation sites of the SUMO ligase PIAS4. When sumoylated, these lysins are masked from UBL2 domain binding and sumoylation was demonstrated to recruit the SUMO-targeted ubiquitin ligase RNF4, which ubiquitinates the TOP-DPCs for subsequent proteasomal degradation [151]. While the annotated fraction of USP7 does not contain predicted docking motifs for the main substrate recognition domain, the MATH domain of USP7, the identified N-terminal extension contains 3 copies of MATH domain docking motifs (2 copies of the type 1 (DOC_USP7_MATH_1) and one copy of the type 2 motif (DOC_USP7_MATH_2), which were reported to bind the same binding groove of the USP7 MATH domain [152]), and thus it is expected to mediate multivalent interactions with USP7 (Figure 6(B)). Therefore, our premise is that the extended TOP1 proteoform could be more efficiently deubiquitinated by USP7 than the normal proteoform.

## Discussion

Differential expression of N-terminally extended proteoforms in cancer cells can be achieved through alterations in certain initiation factors that are involved in start codon stringency. Experiments using reporter constructs and genome-wide CRISPRi screens have shown that dozens of canonical and non-canonical initiation factors can alter initiation at suboptimal start codons in mammals. These ‘stringency’ factors include eIF1, eIF1A, eIF5, BZW1, BZW2, eIF4G2 and eIF3 [19,153–158]. Certain somatic mutations in these ‘stringency’ factors and regulators can lead to altered non-AUG initiation in cancer cells. For example, mutations in the unstructured N-terminal tail (NTT) of *EIF1A* have been associated with uveal melanoma [159], and corresponding mutations in yeast eIF1A’s NTT can suppress initiation at non-AUG codons [160]. Additionally, several somatic mutations in the *EIF4G2* gene have been identified in primary tumours from cancer patients. Some of these mutations affect the binding of the eIF4G2 protein to interacting proteins and its ability to direct mRNA translation [161].

However, as ‘stringency’ factors in general do not seem to be frequently mutated in cancer, other mechanisms may contribute to the altered usage of non-optimal start codons. One possible explanation is the differential protein stability of initiation factors such as eIF1 and eIF5. It has been proposed that the half-life of these proteins differs, and therefore, changes in global protein synthesis or protein degradation can alter their ratio which will result in alterations on start codon selection [162]. Changes in ‘stringency factors’ can also be achieved through modulation of their intracellular localization. Recent study has shown that an increase in the stringency of start-codon selection during mammalian mitosis is mediated by the release of nuclear eIF1 after nuclear envelope breakdown [163]. In addition, post-translational modifications may affect eIF activity. For example, eIF5 can be phosphorylated by casein kinase 2 (CK2), which alters its association with other eIFs. This PTM affects translation initiation [164–167]. However, whether CK2-mediated phosphorylation of eIF5 affects the stringency of start codon selection is still an open question. In summary, deregulation of protein homoeostasis, signalling cascades, and cell cycle progression in cancer cells may lead to differential translation from non-AUG codons.

Finally, it should be noted that the probability of initiation at certain non-AUG codons can critically depend on the mRNA sequences upstream and downstream. Therefore, differential translation of specific N-terminally extended proteoforms can be achieved on a gene-specific basis even without any changes to ‘stringency’ factors [6]. For instance, it has been shown that ribosomal pausing immediately downstream of alternative initiation sites can increase the use of upstream start codons [168]. This pausing is typically relieved by the specialized translation factor eIF5A, and its depletion increases translation from the CUG codon in *MYC* and other transcripts [169]. We expect that the putative N-terminal extensions described in our study may also be under extensive translational control in both normal and cancer cells. This has also been suggested for upstream open reading frames (ORFs) that can impact the main coding sequence and/or produce proteins that contribute to a cancer phenotype [170]. In this study we analysed recently proposed N-terminal extensions of cancer-associated proteins for possible functional readouts. Our analysis highlights that PANTs represent a further layer in protein functional versatility, similar to alternative and tissue-specific splice isoforms [171–173]. We found that the additional sequence regions harbour short linear motifs that confer novel functionalities and regulatory possibilities on the extended proteoforms compared to the canonical ones.

Recent work by Bogaert et al. [174] generated a catalogue of N-terminal extended proteoforms from the cytosol of HEK293T cells and selected 22 N-terminal/canonical protein pairs for interaction mapping using a high-throughput workflow (Virotrap [175]) and yeast two-hybrid screening. From those pairs, three were selected for further experimental validation using affinity purification-mass spectrometry (AP-MS). In agreement with our work, the N-terminal extended proteoforms identified by Bogaert et al. had unique interaction partners when compared to the canonical protein, highlighting the functional diversity created by the presence of the extended proteoforms.

We successfully discovered some very interesting cases where N-terminal extensions (supported by both ribosome footprinting and proteomics evidence) could confer different localization, turnover rates or interactions on proteins classified oncogenic and tumour suppressive. Indeed applications of proteomic approaches are allowing the functional interpretation of proteomics data [176]. Top-down proteomics methods that do not include proteolytic digestion and employ multi-dimensional separation techniques, followed by mass spectrometry [177–179], could be used to comprehensively identify and characterize N-terminally extended proteoforms in different cancers and various cell types. Methods that focus on N-termini, such as COmbined FRActional DIagonal Chromatography (COFRADIC) [180,181], as successfully employed in the work by Bogaert et al. [174], could have expanded use across cancer cell lines. To discover and validate the interactors of N-terminal extensions, AP-MS remains one of the best ways to characterize interactomes [174]; however, it can be labour intensive. Nevertheless, we encourage the scientific community to experimentally validate the discovered interaction sites of the extended proteoforms and elucidate their role in tumorigenesis and cancer progression. The expansion of proteomic methods, in combination with systems and computational biology, will aim to address the vast diversity and dynamics of the proteome and continue to improve our understanding of human biology and disease.

## Methods

### Dataset assembly

Genes encoding proteins with non-annotated N-terminal extensions translated from non-AUG alternative translation initiation sites (TISs) were obtained from the PhyloSET and RiboSET datasets published by Fedorova et al. [51,182]. The two sets were merged to look for an overlap with the list of cancer-associated protein-coding genes downloaded from OncoKB (1164 protein-coding genes as of 8/11/2024) [52,53]. 49 cancer-associated genes could be identified within the merged set of N-terminally extended genes. Of these, 31 belong to RiboSET where the position of the alternative TIS was indicated, thus the extended proteoforms and the extension sequences could be obtained in an automated manner. For the remaining 18 genes belonging to PhyloSET, the N-terminal extension was only predicted by Fedorova *et al*. based on PhyloSCF scores and thus the alternative TISs were not indicated. For these genes, we manually annotated translated N-terminal extensions using RiboCrypt. This manual annotation allowed us to identify translated N-terminal extensions for 9 genes (*TRIM33* (with alternative initiation codon: CUG), *ARID3A* (CUG), *POU2AF1* (CUG), *SPEN* (CUG), *DCUN1D1* (CUG), *UBE2A* (CUG), *MAF* (CUG), *ASXL1* (GUG), *MIB1* (GUG)), while the other 9 genes were removed from the dataset due to the lack of convincing ribosome footprint patterns N-terminal to the annotated TIS. Therefore, we ended up with a list of 40 cancer-associated genes with experimental evidence for an N-terminal extension resulting from an alternative TIS (Table S1), which were subjected to further analysis.

### Computational investigation of the N-terminal extensions

We performed degron and stability predictions on the normal and extended proteoforms of the 40 genes using Degronopedia [54]. The number of predicted degrons, all predicted protein stability index (PSI) scores (N-terminal PSI assuming cleaved initiator Met, N-terminal PSI assuming non-cleaved initiator Met and C-terminal PSI) and the predicted cleavage status of the initiator Met (cleaved/not cleaved (C/NC)) were obtained and compared between the two proteoforms (Table S1).

Prediction of Signal Peptides and their cleavage sites was performed with SignalIP 6.0 [55] web server with the following settings: Organism – Eukarya; Output format – Long output; Model mode – Fast.

The webserver part of the ELM linear motif database [57] was used to obtain predicted short linear motifs (SLiMs) for the extended proteoforms. The predicted linear motifs of the N-terminal extensions (if at least one residue was contributed by the extension sequence to the predicted motif, it was already accepted as an extension motif to be able to identify all possible binding sites contributed or complemented by the extensions) were filtered for those with a pattern probability < 0.015 (this value is provided by the ELM database for annotated motif classes and represents how likely the motif pattern occurs in random sequences) to avoid highly degenerate motifs with excessive false positive prediction rates. For strictly nuclear genes (according to UniProt), the motif search was limited to nuclear motifs, and this was indicated with ‘N!’ in Table S1 column T before listing the detected motifs. The resulting, predicted extension motifs are listed for all 40 genes (Table S1). Subsequently, we performed extensive literature search and mining of protein–protein interaction databases, e.g. BioGRID [183], to identify the subset of the predicted motifs that are likely functional based on matching the functional landscape of the given protein, i.e. providing an explanation for a known functionality or binding partner of the protein that could not be derived from the normal proteoform.

For a subset of the 40 proteins, mainly those undergoing homo-oligomerization or heterodimerization, the AlphaFold 3 (AF3) web server [56] was used to model the potential structural effect of the N-terminal extensions on oligomerization (Table S1). Similarly, AF3 was used to model the selected domain–motif interactions described in the article and depicted in Figures 2–5. In these AF3 predictions, the motifs were provided in the form of peptides that include  ±  5 residues flanking regions around the actual motifs predicted by ELM. Phosphorylated form of the motif was used in the case of phospho-dependent interactions, such as the CKS1 docking motif and the FBXW7 degron motif of SUFU.

### Identification of proteomics evidence for the existence of N-terminal extensions

Sequences of N-terminal extensions including the overlap with corresponding CDS until the first trypsin cleavage site were used as input (SPEN: MTVSYEAGEGEPAAAVAGTPPSMVR; SOX2: MITIIGGGRIGQRRREALFLILIPVCLSLFFPQIILRLIFLAEPCAPDTPARLPSSSPPARGPPKVPAGPRVGGRRRAGPAHSAR; SUFU: MARQCSPRRLPSPVPPVPALRTPMAELRPSGAPGPTAPPAPGPTAPPAFASLFPPGLHAIYGECR; TOP1: MRLLEPPESPSARTGRFAVCVSPTPPRLPPRSSLRADMSGDHLHNDSQIEADFR; SFPQ: MASTFPERLLRFCLDRPLTTDM SR;) we searched for proteomic evidence using targeted peptide search engine PepQuery2 [83]. PepQuery analysis was conducted using a web-based application with the following default settings: PepQuery version 2.0.2, Fixed modification -Carbamidomethylation of C, Variable modification – Oxidation of M, Maximal allowed variable modification  −  3, Add AA substitution – false, Enzyme – Trypsin, Max Missed cleavages  −  1, Precursor mass tolerance  −  20.0, Range of allowed isotope peak errors  −  0, Precursor ion mass tolerance unit – ppm, Fragment ion mass tolerance  −  0.6, Fragment ion mass tolerance unit – Da, Scoring algorithm – Hyperscore, Min score  −  12.0, Min peaks  − 10, Min peptide length  − 7, Max peptide length  − 45, Min peptide mass500.0, Max peptide mass  −  10000.0, Random peptide number  −  1000 in fast searching mode. The reference database used is Gencode_V34_human. The following MS/MS datasets identifiers were used for analysis: PDC000220, PDC000219, MSV000085836, PDC000128, PDC000127, PDC000245, PDC000205, PDC000204, PDC000222, PDC000221, PDC000233, PDC000232, PDC000234, PDC000237, PDC000224, PDC000149, PDC000153, PDC000271, PDC000270, PDC000176, PDC000180, PDC000239, PDC000121, PDC000120, PDC000117, PDC000116, PDC000109, PDC000251, PDC000110, PDC000119, PDC000118, PDC000174, PDC000173, PDC000111, PDC000112, PDC000115, PDC000114, PDC000226, PDC000126, PDC000125, PDX010154, PDX016999, PDC000198, PDC000262, PDC000216, PDC000215, PDC000214.

### Software tools used for visualizations

Figure panels showing domain maps were created using DOG2 visualization software [184], while AF3-predicted structural models were depicted using UCSF ChimeraX [185]. The Venn diagram was drawn using a dedicated website: https://bioinformatics.psb.ugent.be/webtools/Venn/.

## Supplementary Material

Supplemental Material

## Data Availability

Data for this publication is contained in the paper and the supplementary material.
